# Metabolic Profiling of Chestnut Shell (*Castanea crenata*) Cultivars Using UPLC-QTOF-MS and Their Antioxidant Capacity

**DOI:** 10.3390/biom12121797

**Published:** 2022-12-01

**Authors:** Miso Nam, Ja Myung Yu, Young Ran Park, Young-Sik Kim, Jae-Ho Kim, Min-Sun Kim

**Affiliations:** 1 Food Analysis Research Center, Korea Food Research Institute, Wanju 55365, Republic of Korea; 2 Korea Health Supplements Association Sub. Korea Health Supplements Institute, Seongnam-si 13488, Republic of Korea; 3 Department of Herbology, College of Korean Medicine, Woosuk University, Jeonju 55338, Republic of Korea; 4 Enterprise Solution Research Center, Korea Food Research Institute, Wanju 55365, Republic of Korea

**Keywords:** metabolic profiling, *Castanea crenata* cultivar, chestnut shell, antioxidant capacity, mass spectrometry

## Abstract

The inner shell of the chestnut (*Castanea crenata*) has long been used in Asia as a medicinal herb for improving digestion and blood circulation, and treating diarrhea. However, most chestnut shells are now treated as waste materials in industrial peeling processes. In this study, we examined the metabolite variation among major cultivars of *C. crenata* shells using mass spectrometry. Among five representative cultivars, Okkwang, Porotan, and Ishizuuchi had higher levels of bioactive compounds, such as ellagic acid derivatives, ellagitannins, flavonoids, and gallic acid derivatives. Their antioxidant capacity was positively correlated with their chemical composition. The byproducts (whole shells) from the industrial peeling process were re-evaluated in comparison with the inner shell, a rich source of phenolic compounds. The phenolic acids and flavonoid glucoside derivatives were significantly higher in the whole shells, whereas the levels of flavonoids were higher in the inner shells. In addition, the whole shell extracts significantly reduced cellular reactive oxygen species production compared to the inner shell extracts. This study demonstrated the different biochemical benefits of different *C. crenata* cultivars through metabolic profiling and suggests that the whole shell could be used as a functional ingredient, as it has the highest levels of bioactive products and antioxidant effects.

## 1. Introduction

Chestnut (*Castanea crenata Siebold & Zucc*., Okkwang, Daebo, Riheiguri, Porotan, and Ishizuuchi) belongs to the *Fagaceae* family that grows wild throughout Korea and Japan. They are mainly used for food, especially processed foods, and in medicine. In 2019, the FAO (Food and Agriculture Organization of the United Nations) reported that 2,321,780 tons of chestnuts were produced globally [1]. Korea is the second-largest chestnut producer in Asia, producing 50,000 tons of chestnuts annually [2]. About 45% of Korean chestnuts are exported as peeled, canned, and raw chestnuts [3]. The integument of chestnuts consists of a hard hull (outer shell) and thin skin (inner shell), which make up 15–20% of the fresh fruit weight [1,4]. During industrial chestnut peeling processes, the shells are considered solid waste and thrown away. However, they remain a useful source of bioactive compounds [1,5]. The chestnut inner shell, in particular, has been used for many years in traditional medicine in Asian countries and is known for its antioxidant, antiallergic, antidiabetic, and anti-amnesic properties [6,7,8,9]. Several studies have demonstrated the biological activities of the *Castanea* genus, including antioxidant and anticancer activities [10,11,12]. These positive effects on human health and wellness are closely related to the total phenols, tannins, and flavonoids [4,13]. The nutritional composition of the shells of Portuguese, Spanish, and Italian *C. sativa* cultivars and their antioxidant capacity have been determined [4,14,15]. However, no reports on the metabolite composition of *C. crenata* shells were found in the literature. We applied metabolomic analysis to investigate the difference in the chemical composition of chestnut shell extracts. As plants produce a large spectrum of compounds with diverse chemical properties, a metabolomics approach is necessary for coverage of a broad range plant metabolites [16,17]. Mass spectrometry (MS) combined with chromatography has a high mass resolution and accuracy, in both MS profiling and MS/MS experiments for the qualitative and quantitative analysis of a wide array of metabolites [18,19]. There have not been many metabolomics studies on chestnuts shells, and the metabolite composition of *C. crenata* shells using quadrupole time-of-flight MS (QTOF/MS) has not been reported to date.

In 1958, the chestnut gall wasp (*Drycocosmus kuriphylus Yasumatsu*) first appeared in Korea and spread nationwide, damaging and killing native chestnut trees. Since then, large-scale chestnut tree hybridization has been used to develop highly productive cultivars resistant to pests and diseases [20]. The commonly cultivated chestnuts in Korea are derived from intra-hybridization with Japanese chestnut trees or individual selection. Currently, in Korea, cultivars native to Japan or Korea, such as Okkwang, Daebo, Ishizuuchi, Porotan, and Riheiguri, are widely cultivated [21].

This work investigated the difference in the chemical composition of chestnut shell extracts from *C. crenata* cultivars. Knowing which chestnut cultivars exhibit the highest level of bioactive compounds will be beneficial for selecting better chestnut cultivars. We also examined the different metabolite levels in extracts of the inner and whole shells, to evaluate the value of the whole shells discarded as industrial waste, from both economic and environmental points of view. Our study (a) characterized a variety of metabolites in *C. crenata* shells with exact mass values and MS/MS spectra of native and hybrid cultivars using ultra-high performance liquid chromatography (UPLC)–QTOF/MS, (b) quantified important metabolites in the inner and whole shells with liquid chromatography-quadrupole/linear ion trap mass spectrometry (LC-QTRAP/MS), and (c) interpreted the relationship between the chemical composition and strong antioxidant capacity.

## 2. Materials and Methods

### 2.1. Chemicals

Methanol, water, and acetonitrile of LC-MS grade were purchased from Honeywell (St. Muskegon, MI, USA). Formic acid was obtained from Fisher Scientific (Pittsburgh, PA, USA). All authentic standards were bought from Sigma Chemical (St. Louis, MO, USA).

### 2.2. Plant Materials

Chestnuts from five cultivars (Okkwang, Daebo, Riheiguri, Porotan, and Ishizuuchi) of *C. crenata* were purchased fresh from local chestnut processors and local distributors between September and November 2020, from local markets in Republic of Korea. To ensure that variety-identified chestnuts were collected, the acquisition was made through local contacts. Figure S1 shows the samples photographed before being peeled and dried. The sample information of *C. crenata* are shown in Table S1. Fresh fruits were maintained under vacuum at −1 °C until extraction.

The whole shells (outer and inner shell) from chestnuts were automatically separated using a chestnut skin removal machine. The inner (which directly covers the kernel) shells were separated from the fruits by hand for the analysis. The samples were prepared using a freeze-drying process under 10 mTorr at −50 °C for 72 h using a FDCF-12003 freeze dryer (Operon, Gimpo-si, Republic of Korea). Dried shells were ground and stored at −80 °C until further use.

### 2.3. Sample Preparation

Freeze-dried chestnut shells (30 mg) were extracted using 1 mL ethanol/water (70:30, *v/v*). The sample was ultrasonicated for 20 min. The supernatant solution was filtered through 0.22 μm PTTE membranes (Millipore, Billerica, MA, USA) before UPLC-QTOF/MS and LC-QTRAP/MS analysis.

### 2.4. Mass Spectrometry Analysis and Data Processing

All UPLC-QTOF/MS analyses were performed using a X500R QTOF system coupled to an Exion LC™ AD system (SCIEX, Concord, ON, Canada). The analysis conditions were as follows: Acquity UPLC HSS T3 column (2.1 mm × 100 mm, 1.7 μm; Waters); solvent system, 0.1% formic acid in water (phase A), and 0.1% formic acid in acetonitrile (phase B); gradient program, 1–5% B from 0 to 1 min, 5–25% B from 1 to 3 min, 25–35% B from 3 to 4.8 min, 35% B from 4.8 to 5.8 min, 35–45% B from 5.8 to 6.8 min, 45% B from 6.8 to 7.8 min, 45–60% B from 7.8 to 8.8 min, 60–100% B from 8.8 to 9.3 min, and then back to the initial condition from 9.3 to 13 min; flow rate, 0.45 mL/min; temperature, 40 °C; and injection volume, 5 µL.

The chestnut shell samples were analyzed both in positive and negative ionization modes and the mass range was from 50 to *m*/*z* 1100. In positive and negative mode, the ion spray voltage was set at 5500 V and −4500 V, the curtain gas at 30 psi, nebulizing (GS1) at 50 psi; heating (GS2) at 60 psi, and the source temperature at 500 °C. The IDA function was performed, in which the five most intense mass peaks were fragmented. Sciex O.S. software V 1.5 (SCIEX) was employed for data acquisition and processing.

For targeted analysis, 14 selected compounds were quantified using LC-QTRAP/MS-MRM mode. LC separation was implemented using a Syncronis C18 column (2.1 mm × 100 mm, 3 μm; Thermoscientific) at a flow rate of 0.3 mL/min. A linear gradient from 1 to 50% B in 6 min, from 50 to 99% B in 1 min, held at 99% B for 1 min, from 99 to 1% B in 1 min, and held at 1% B for 5 min was used. The injection volume was 5 μL. The MRM transitions and retention times of the metabolites are summarized in Table S2.

### 2.5. In Vitro Activities of the Chestnut Shell Extracts

#### 2.5.1. Preparation of the Extracts

Approximately 50 mg of dried shell and inner shell were extracted with 1 mL of ethanol/water (70:30, *v*/*v*). The sample was vortexed for 2 min and sonicated for 1 h at room temperature. After centrifugation (12,000 rpm, 20 min, room temperature), the supernatants were filtered through a 0.45 μm PTFE syringe filter (Millipore). The filtered extracts were prepared using a freeze-drying process under 10 mTorr at −50 °C for 72 h using a FDCF-12003 freeze dryer (Operon, Republic of Korea). Dried extracts were stored at −80 °C until further use.

#### 2.5.2. Total Phenolic Content (TPC)

The total phenolic content (TPC) was determined by the modified Folin–Ciocalteu method [22]. The extracts (0.5 mg/mL), diluted with ethanol/water (70:30, *v*/*v*), were mixed with 0.2 N Folin–Ciocalteu reagent for 6 min and then reacted with 10% sodium carbonate solution for 1 h. Then, the absorbance was read at 765 nm using a Synergy H1 microplate reader (Bio Tek, Winooski, VT, USA). The phenolic content in the extract of whole shells was shown as milligram of gallic acid equivalents (GAE) per 100 g of dry extract of each sample (mg GAE/100 g dry weight).

#### 2.5.3. 2,2-Diphenylpicrylhydrazyl (DPPH) Radical Scavenging Assay

The DPPH free-radical scavenging activity of whole shell extracts (0.1 mg/mL) was estimated as described in [22], with some modifications. DPPH (0.2 mmol/L, 100 μL) solution was added to a 96 well plate and 100 μL of different concentrations of sample solution was added.

The mixture was quenched and left at room temperature for 5 min in a darkroom. The absorbance was then measured at 517 nm with a microplate reader. The DPPH radical-scavenging activity (RSA) was calculated using the following equation: % RSA = [(A_control_ − A_sample_)/A_control_] × 100, where A_control_ and A_sample_ are the absorbance. Results are shown as half-minimal inhibitory concentrations (IC_50_) calculated by comparing RSA graphs against extract concentrations.

#### 2.5.4. Ferric Reducing Antioxidant Power (FRAP) Assay

A FRAP assay was performed using a MAK369-1KT kit (Sigma, USA). The procedure was carried out in accordance with the manufacturer’s instructions. Samples (0.2 mg/mL) were added to a 96 well plate, then assay buffer and working solution were added and incubated at 37 °C for 1 h. Absorbance was measured at a wavelength of 594 nm using a microplate reader.

#### 2.5.5. Determination of Intracellular ROS Scavenging Activity

GES1 cells were purchased from the Korea Cell Line bank (KCLB, Seoul, Republic of Korea). The cells were grown at 37 °C in humidified 5% CO_2_, 95% air mixture in Dulbecco’s modified Eagle’s medium (DMEM) supplemented with 10% fetal bovine serum (FBS), 100 U/mL of penicillin, and 100 μg/mL of streptomycin.

Intracellular ROS levels were measured using a DCF-DA assay [23]. Briefly, GES1 cells were seeded in a black 96 well plate (2 × 10^4^ cells/well) and pre-incubated with 10 μM DCF-DA (dissolved in DMSO) for 45 min at 37 °C in the dark. After washing the excess probe using phosphate-buffered saline (PBS), the cells were treated with DW for the control group, 6 mM N-acetylcysteine amide (NAC) (positive control), or extracts (100 μg/mL) of shells in the presence of 2 mM H_2_O_2_ for 4 h, and then washed with PBS. Fluorescence was detected using a microplate reader using a λ (ex./em.) = 485/535 nm.

### 2.6. Statistical Methods

To verify differences between more than two groups, a Kruskal–Wallis test followed by Dunn’s post hoc test was performed using SPSS 12.0 software (SPSS Inc., Chicago, IL, USA). A heat map and hierarchical cluster analysis (HCA) were generated using MetaboAnalyst Ver. 5.0.

## 3. Results and Discussion

In this study, representative chestnut cultivars (Okkwang, Daebo, Riheiguri, Porotan, and Ishizuuchi), originating from Buyeo-gun or Gongju-si, South Chungcheong Province, the main chestnut-producing regions in Korea, were used to determine the metabolite differences among *C. crenata* cultivars. Their origin and crossbreeding information are shown in Table S1.

### 3.1. The Metabolic Composition of Whole C. crenata Shells

To evaluate the differences in chemical composition between *C. crenata* cultivars, non-target metabolite profiling of chestnut extracts was performed using UPLC–QTOF/MS. The metabolites were tentatively identified with the exact mass values of the precursor and the MS/MS spectral data of authentic standards, previously published data, or the MassBank and METLIN databases [24]. The total ion chromatograms (TIC) of the chestnut extracts are given in Figure S2. The characteristic ions corresponding to each compound are showed in Table 1. The mass accuracy of the [M+H]^+^ or [M−H]^−^ ions was within ±8.89 ppm.

The primary compounds identified from the *C. crenata* shell extracts were ellagitannins. Ellagitannins are hydrolyzable tannins containing polyphenolic compounds and a sugar core [25]. These compounds protect against oxidative stress-related diseases and prevent degenerative diseases, such as cardiovascular and inflammatory diseases and cancers [4,11,13]. Each compound was tentatively characterized through loss of a hexahydroxydiphenyl (HHDP), a sugar unit, and gallic acid moieties. The predominant ion at *m*/*z* 301 was formed from the ellagic acid anion produced by the rearrangement of the HHDP moiety [5]. Bis-HHDP-glucose showed [M−H]^−^ ion at *m*/*z* 783 and fragment ions at *m*/*z* 765 [M-H-H_2_O]^−^, 481 [M-H-HHDP]^−^, and 275 [M-H-HHDP-glucose-CO_2_]^−^. Tri-, di-, and galloyl-HHDP-glucoside produced [M−H]^−^ ions at *m*/*z* 937, 785, and 633. The most characteristic ions of galloyl-HHDP-glucosides are matched by the loss of a gallolyl unit at *m*/*z* 463 and 300, corresponding to [M-H-Gal-glucose]^−^ [26].

A valoneoyl group consists of an HHDP group and a galloyl moiety connected by a C–O–C bond [27]. In the MS/MS experiment, HHDP-valoneoyl-glucose exhibited fragment ions at *m*/*z* 907 and 783, which corresponded to the loss of CO_2_ ([M−COOH]^−^) and a gallolyl unit from the [M−H]^−^ ion, respectively. NHTP (nonahydroxytriphenoyl-) glucose derivatives are based on a core of glucose esterified with HHDP or NHTP groups [28].

NHTP-HHDP-glucose showed a [M−H]^−^ ion at *m*/*z* 933 and fragment ions at *m*/*z* 915 [M-H-H_2_O]^−^, 631 [M-H-HHDP]^−^, and 425 [M-H-HHDP-glucose-CO_2_]^−^ in the MS/MS experiment. Ellagitannins were also reported in *C. sativa* Mill. shells in a previous study, and they possessed a wide range of biological activities [5].

In agreement with a previous report, various proanthocyanidins were detected in chestnuts [29]. The highly enriched proanthocyanidins showed significant anti-inflammatory activity. Proanthocyanidins are condensed tannins found in the nuts, bark, fruits, and seeds of various plants [30] and are anti-inflammatory and have beneficial effects on metabolic syndrome, atherosclerosis, and cancer [29]. Proanthocyanidins are formed by catechins and epicatechins [31]. These compounds produced the same fragment ions at *m*/*z* 289, 303, and 305 in the MS/MS experiment, suggesting that these compounds were composed of either catechin (C) or gallocatechin (GC) aglycones [32]. The loss of 170 Da or 188 Da (170 + 18) was caused by the release of a gallic acid and a water molecule.

Flavonoids are one of the major classes of phenylpropanoids abundantly found in various foods and beverages [33]. Flavonoids were tentatively identified in the chestnut shells, including flavonol, flavone, flavanone, and their sugar conjugates. Kaempferol showed the [M+H]^+^ ion at *m*/*z* 287 in the positive ion mode, with a well-shaped peak observed for kaempferol derivatives. Kaempferol coumaroyl hexose was confirmed by the identification of the fragment ion at *m*/*z* 309 via the loss of a kaempferol moiety from [M+H]^+^. Luteolin displayed the same [M+H]^+^ ion as kaempferol at *m*/*z* 287, confirmed by comparing their retention times and MS/MS spectra with the relevant standards. The most abundant ions of quercetin derivatives at *m*/*z* 301 represented the loss of a sugar moiety. Rutin is characterized using the fragment ions at *m*/*z* 301 and 273 in negative mode, which result from the loss of the rhamnose-glucose unit and the serial loss of CO, respectively.

Myricetin, isorhamnetin, and apigenin were detected at *m*/*z* 317, 315, and 269 from [M−H]^−^. The flavanones eriodictyol and naringenin were tentatively identified by the presence of *m*/*z* 289 and 273 ions in positive mode. The most characteristic ion of these compounds at *m*/*z* 151 (negative mode) or 153 (positive mode) were generated by *retro*-Diels–Alder fragmentation [34]. Myricetin-hexoside showed the loss of the glucose unit (*m*/*z* 479 → *m*/*z* 317) in negative mode. Naringin and naringenin glucosides were identified using the predominant ion at *m*/*z* 271 from the aglycone of naringenin in negative mode.

Ellagic acid is a phenolic acid found in various fruits and vegetables, with antioxidant and antiviral properties [35,36]. Ellagic acid derivatives, such as ellagic acid hexose, -pentose, and -deoxyhexose, yielded the predominant fragment ion at *m*/*z* 301 in negative mode from an ellagic acid moiety. Methylated ellagic acids were generated as methyl, dimethyl, and trimethylellagic acid at *m*/*z* 315, 329, and 243, respectively, by the addition of methyl groups to ellagic acid.

Gallic acid and derivatives showed high antioxidant activity and may play protective roles, including anticancer, antiviral, antifungal, and antibacterial activities [37]. Gallic acid displayed [M−H]^−^ ions at *m*/*z* 169 and an abundant fragment ion at *m*/*z* 125, due to the loss of carboxylic acid. Digalloyl-, trigalloyl-, and tetragalloyl glucose were characterized by a product ion at *m*/*z* 465, which could be attributed to the loss of H_2_O and gallic acid moieties from the [M−H]^−^ ion.

### 3.2. Differences in Metabolite Levels Associated with Whole C. crenata Shells

To assess the differences in the chemical compositions of the *C. crenata* cultivars, principal component analysis, specifically partial least squares discriminant analysis (PLS-DA), was performed on the mass spectra. The PLS-DA score plots derived from the positive (Figure S3A) and negative (Figure S3B) modes showed a significant separation in *C. crenata* cultivars (positive, *R*^2^ = 0.547, *Q*^2^ = 0.371; negative, *R*^2^ = 0.770, *Q*^2^ = 0.711). The five *C. crenata* cultivars in this study were clustered in both polarity modes. Okkwang, Porotan, and Ishizuuchi were separated from the other cultivars, Daebo and Riheiguri, which formed a separate group. These results showed the close relationship due to their origins. Daebo originated by crossbreeding with “Sangmyeon,” a Korean native cultivar, and Riheiguri (Table S1).

**Figure 1:**
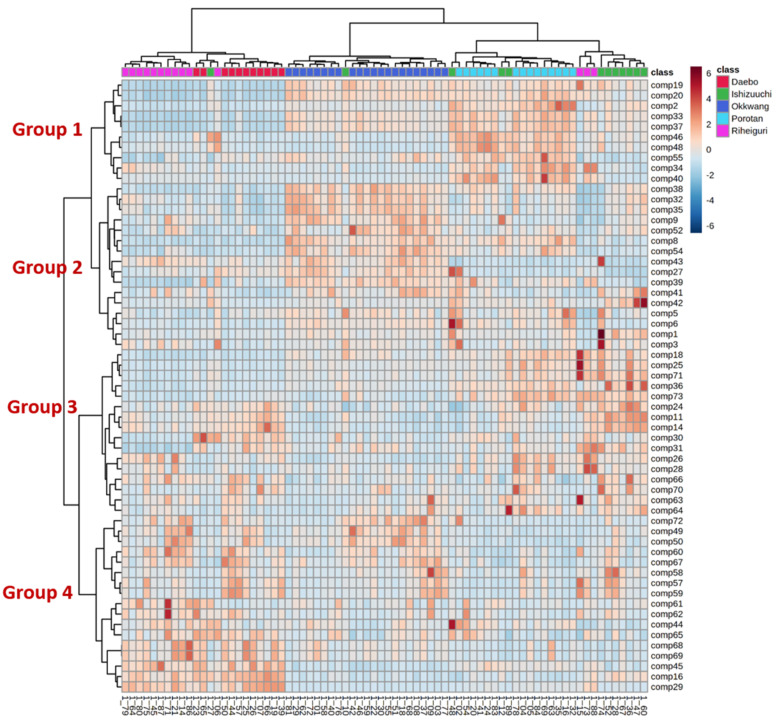
Heatmap visualization of the metabolites identified in 74 *C. crenata* whole shell samples from Daebo, Riheiguri, Okkwang, Porotan, and Ishizuuchi. For interpretation of the compound numbers in this figure, the reader is referred to Table 1. (For statistical analysis of the metabolites among cultivars, duplicate compounds with the same pattern were excluded).

**Figure 2:**
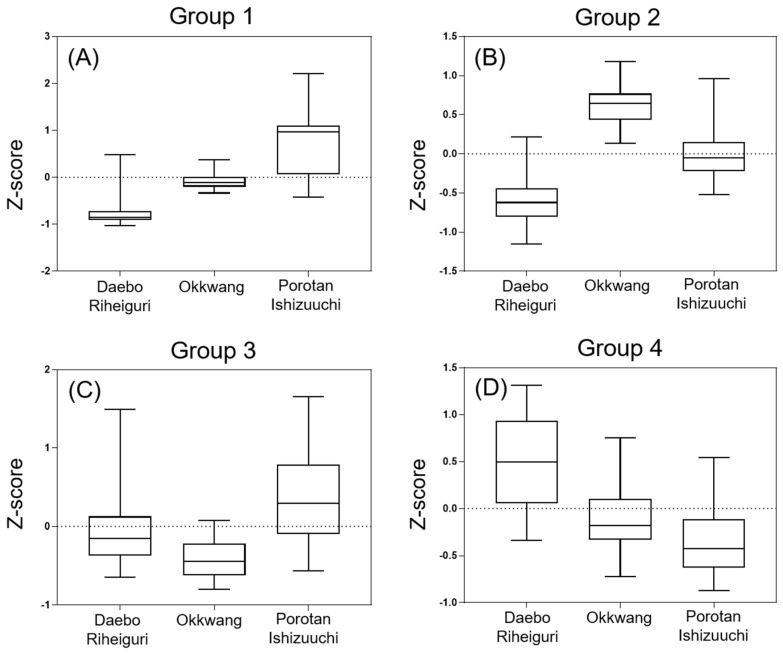
Boxplots of the relative contents of each group of compounds in the major *C. crenata* cultivars. (**A**) Group 1, (**B**) Group 2, (**C**) Group 3, and (**D**) Group 4. The Z-score data in this figure were extracted from Figure 1.

In order to visualize the relationship of the differential metabolites identified in the *C. crenata* cultivars, a heatmap visualization was employed (Figure 1). In Figure 1, the chestnut shell samples were divided into three branches according to their cultivars: Daebo and Riheiguri formed one group, Okkwang formed another group, and Porotan and Ishizuuchi formed a final group. The differential compounds were separated into four groups, which indicated that the identified metabolites could be used to identify the cultivars of chestnuts from their shells. The identified metabolites included in each group are shown in Table S3. To obtain an insight into the behavior of these metabolites in chestnut shells from different cultivars, the relative contents of each group of metabolites are displayed as boxplots in Figure 2. The Group 1 compounds are most abundant in Porotan and Ishizuuchi, which contain high levels of flavonols and methylellagic acids. The Group 2 compounds, which were higher in Okkwang, are mainly ellagitannins and ellagic acid glucosides. Most ellagic acid is present as hydrolyzable ellagitannin in the vacuoles of plant cells, and ellagitannins produce ellagic acid during hydrolysis of the HHDP group [38]. In this study, the level of ellagic acid was positively correlated with ellagitannins. The compounds in Group 3, which are mainly flavonoids and proanthocyanidins, had a high degree of variation within the samples in each group. Amino, organic, and phenolic acids were the dominant compounds in Group 4 and were abundant in Daebo and Riheiguri.

To determine the relationship between the metabolite contents and the antioxidant effects, the total phenolic content and the antioxidant capacity of the *C. crenata* shell extracts were measured. The results of the total phenolic content, DPPH radical scavenging assay, and FRAP assay are shown in Table 2. The antioxidant effects of the *C. crenata* shell extracts determined using the DPPH and FRAP assays were correlated with the total phenolic content. The Okkwang, Porotan, and Riheiguri extracts presented higher antioxidant activities than the Daebo and Riheiguri extracts, similarly to the results obtained from the metabolite profiling of *C. crenata*. In previous reports, differences in the chemical composition and antioxidant capacities among chestnut (*C. sativa* Mill. and *C. mollissima*) cultivars were observed [5,39,40,41].

### 3.3. Metabolite Quantification in Inner and Whole Shells of C. crenata

Chestnut inner shells are a rich source of total phenols and hydrolyzable tannins and flavonoids [10,42], but they are separated from the kernel with the outer shell during industrial peeling processes and are considered waste materials. To separate only the inner shell of the chestnuts is a difficult, time-consuming, and labor-intensive process. To re-evaluate the effective use of whole shells, the metabolic composition and antioxidant capacities of the inner and whole shells of chestnuts were compared.

To investigate the contribution of the different parts of the shells to the phytochemical composition and biological activity, chestnut extracts from the inner parts and whole shells were prepared separately, as described in Section 2.2. The metabolic profiling data showed that bioactive compounds, such as phenolic acid derivatives, flavonoids, tannins, and proanthocyanidins, had the highest intensities in the Okkwang, Porotan, and Ishizuuchi cultivars (Figure 1). Thus, these three cultivars were selected to determine the differences between the inner and whole shells of *C. crenata*.

A targeted analysis was performed on an LC-QTRAP/MS, to determine the quantities of differential metabolites in the inner and whole shells. Targeted analyses of *C. crenata* shell samples were performed for the phenolic acids, namely caffeic acid, ferulic acid, gallic acid, shikimic acid, chlorogenic acid, and coumaric acid; the flavonoids, namely catechin, quercetin, quercetin glucoside, rutin, apigenin, luteolin, and naringenin; and the amino acids, namely tryptophan. To quantify the metabolites, the phenylalanine-^13^C_6_ were used as internal standards, improving the quantification’s precision and accuracy.

**Figure 3:**
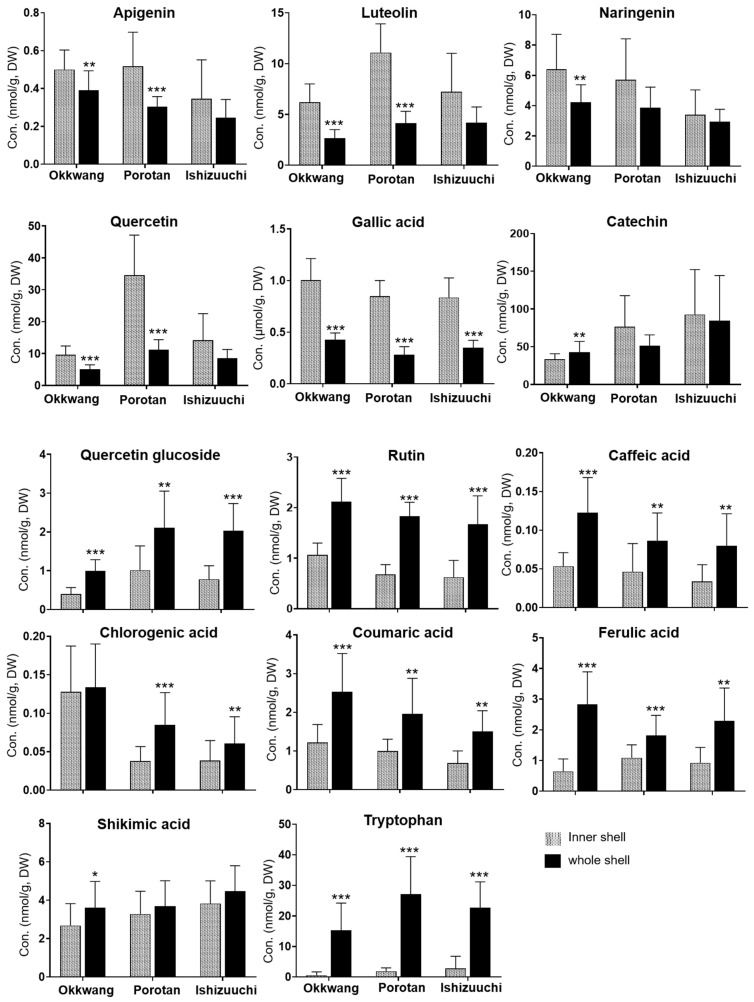
Variation in metabolite levels in inner and whole shells of *C. crenata* cultivars. Vertical lines indicate standard deviation (Okkwang, n = 22; Porotan, n = 15; Ishizuuchi, n = 12). Significance levels on pairwise comparisons of the inner and whole shell samples are defined as * *p* < 0.05, ** *p* < 0.01, *** *p* < 0.001.

The calibration standards were generated using a solution of combined standards for the appropriate range of each compound. The equations for the calibration curves and the linear regression coefficients for the targeted metabolites are given in Table S4. The calibration curves were constructed using a linear least squares regression analysis of the analyte and internal standard. The calibration curves of the compounds showed correlation coefficients (*R*^2^) greater than 0.99 within the given concentration ranges. Figure 3 shows the difference in the levels of significant metabolites in the different parts and cultivars of chestnut shells.

The inner shell extracts contained higher levels of flavonoids, including apigenin, luteolin, naringenin, and quercetin, than the whole shell extracts. In comparison, the whole shell extracts showed predominant concentrations of flavonoid glucosides, such as quercetin glucoside and rutin. The phenolic acids, including caffeic acid, chlorogenic acid, coumaric acid, ferulic acid, and shikimic acid, were more abundant in the whole shell extracts. However, the inner shell extracts contained more gallic acid. The distribution of catechin in the inner and whole shell extracts differed for each cultivar; in particular, tryptophan was barely detected in the inner shell extracts.

**Figure 4:**
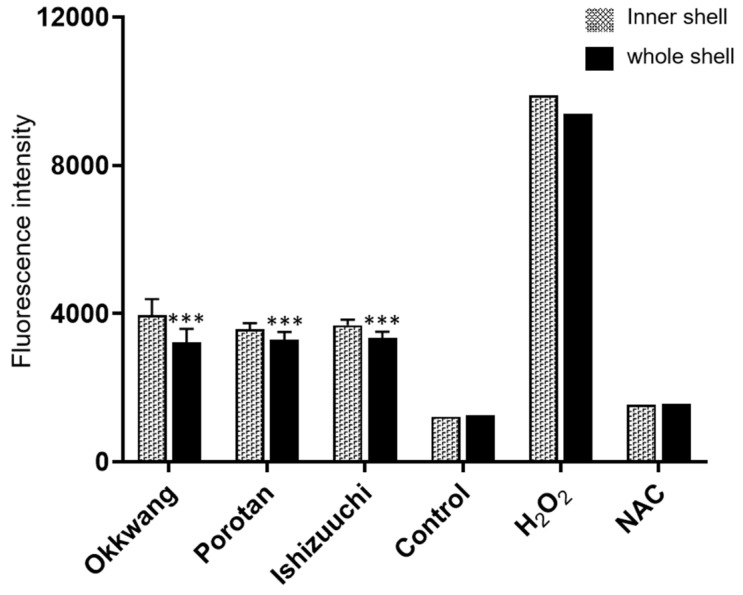
Antioxidant activity of *C. crenata* shell extracts determined using an in vitro GES1 cell-based assay. Vertical lines indicate standard deviations (Okkwang, n = 22; Porotan, n = 15; Ishizuuchi, n = 12). Significance levels on pairwise comparisons of the inner and whole shell samples are defined as *** *p* < 0.001.

In a previous study of the potential anti-gastritis properties of chestnut (*C. sativa* Mill.), chestnut flour was devoid of any anti-inflammatory activity, while the inner and outer shells retained the ability to inhibit IL-8 secretion. In addition, it has reported that the inhibitory activity on IL-8 secretion was highly similar between the inner and outer shells [29]. We utilized a GES1 cell-based assay to evaluate the ability of the inner and whole shell extracts of *C. crenata* to reduce the cellular reactive oxygen species (ROS) production stimulated by H_2_O. The intracellular levels of ROS generated after stimulation of the GES1 cells with H_2_O_2_ were significantly reduced, by 100 μg/mL, for both the inner and whole shell extracts (Figure 4). The whole shell extracts more significantly reduced the intracellular levels of ROS than the inner shell extracts. In summary, the whole shells produced by mechanical peeling had as much antioxidant and biological activity as the inner shells.

## 4. Conclusions

In the present study, we determined the metabolite profile of chestnut shells from different cultivars of *C. crenata* using UPLC–QTOF/MS. The identification of metabolites was confirmed using high-resolution MS/MS analysis, complemented by authentic standards and the calculation of the corresponding molecular formulas. We found that bioactive compounds, such as ellagic acid derivatives, ellagitannins, flavonoids, proanthocyanidins, and gallic acid derivatives, were more abundant in the Okkwang, Porotan, and Ishizuuchi cultivars. Corresponding higher antioxidant activities were observed in these cultivars using the DPPH and FRAP assays. Our study suggests the biochemical benefits of *C. crenata* shells, which can help growers and consumers intentionally choose chestnut cultivars.

To investigate the contribution of the inner and whole shells to the biological activity, several phenolic compounds were quantified using MRM transitions. The concentrations of the flavonoids were higher in the inner shells; however, the levels of phenolic acids and flavonoid glucoside derivatives were higher in the whole shells. The whole shell extracts significantly reduced the intracellular levels of ROS compared to the inner shell extracts, indicating that the whole shell has as much biological activity as the inner shell and represents a valuable new source of bioactive phytochemicals.

## Figures and Tables

**Table 1:** Compounds detected using UPLC-QTOF/MS in whole *C. crenata* shells.

No	Compound	Rt (min)	Ionization Mode	Molecular Formula	Observed Precursor Ions (*m*/*z*)	Difference (ppm)	Product Ions (*m*/*z*)
	*Ellagitannins*						
1	HHDP-glucose	2.02	[M−H]^−^	C_20_H_18_O_14_	481.0627	1.87	300, 275
2	Galloyl-HHDP-glucose	2.75	[M−H]^−^	C_27_H_22_O_18_	633.0721	−0.95	481, 463, 300, 275
3	NHTP-HHDP-glucose	2.76	[M−H]^−^	C_41_H_26_O_26_	933.0625	−0.96	915, 631, 613, 425, 301
4	Bis-HHDP-glucose	2.91	[M−H]^−^	C_34_H_24_O_22_	783.0662	−2.43	765, 481, 301, 275
5	Bis-HHDP-glucose	2.93	[M−H]^−^	C_34_H_24_O_22_	783.0674	−0.89	765, 481, 301, 275
6	HHDP-valoneoyl-glucose	3.17	[M−H]^−^	C_41_H_28_O_27_	951.0723	−1.68	907, 783, 465, 301
7	Galloyl-HHDP-glucose	3.20	[M−H]^−^	C_27_H_22_O_18_	633.0718	−1.42	481, 463, 300, 275
8	Digalloyl-HHDP-glucose	3.62	[M−H]^−^	C_34_H_26_O_22_	785.0829	−1.02	633, 615, 463, 301, 275
9	Trigalloyl-HHDP-glucose	3.95	[M−H]^−^	C_41_H_30_O_26_	937.0946	−0.11	767, 633, 617, 465, 301
	*Proanthocyanidins* ^a^						
10	GC-GC-C B-type trimer	2.47	[M−H]^−^	C_45_H_38_O_20_	897.1866	−1.34	729, 711, 425, 407, 303, 289
11	GC-GC-C B-type trimer	2.61	[M−H]^−^	C_45_H_38_O_20_	897.1861	−1.89	729, 711, 425, 407, 303, 289
12	GC-GC-GC B-type trimer	2.62	[M−H]^−^	C_45_H_38_O_21_	913.1799	−3.07	727, 559, 423, 305, 303
13	GC-GC-GC B-type trimer	2.67	[M−H]^−^	C_45_H_38_O_21_	913.1822	−0.55	727, 559, 423, 305, 303
14	GC-GC B-type dimer	2.76	[M−H]^−^	C_30_H_26_O_14_	609.1232	−1.97	591, 483, 441, 423, 305
15	C-C-C B-type trimer	2.82	[M−H]^−^	C_45_H_38_O_18_	865.1974	−0.58	847, 713, 695, 577, 425, 407, 289, 287
16	GC-GC-GC B-type trimer	2.89	[M−H]^−^	C_45_H_38_O_21_	913.1827	0.00	727, 559, 423, 305, 303
17	GC-GC B-type dimer	3.16	[M−H]^−^	C_30_H_26_O_14_	609.1238	−0.99	591, 483, 441, 423, 305
18	C-C B-type dimer	3.24	[M−H]^−^	C_30_H_26_O_12_	577.1345	−0.17	425, 407, 289, 245, 125
19	C-C-C B-type trimer	3.46	[M−H]^−^	C_45_H_38_O_18_	865.1967	−1.39	847, 713, 695, 577, 425, 407, 289, 287
20	C(G)-C B-type dimer	3.77	[M−H]^−^	C_37_H_30_O_16_	729.1446	−1.23	577, 559, 451, 425, 407, 289, 287
21	C-C B-type dimer	3.78	[M−H]^−^	C_30_H_26_O_12_	577.1349	0.52	425, 407, 289, 245, 125
22	C-C B-type dimer	3.95	[M−H]^−^	C_30_H_26_O_12_	577.1341	−0.87	425, 407, 289, 245, 125
23	C(G)-C B-type dimer	4.36	[M−H]^−^	C_37_H_30_O_16_	729.1487	4.39	577, 559, 451, 425, 407, 289, 287
	*Flavonoids*						
24	Epigallocatechin	2.93	[M−H]^−^	C_15_H_14_O_7_	305.0652	−2.95	261, 179, 167
25	Catechin *	3.50	[M−H]^−^	C_15_H_14_O_6_	289.0717	1.73	245, 221, 203
26	Myricetin hexose	4.03	[M−H]^−^	C_21_H_20_O_13_	479.0833	1.67	317
27	Rutin *	4.22	[M−H]^−^	C_27_H_30_O_16_	609.1472	2.79	301, 273
28	Quercetin glucoside *	4.35	[M−H]^−^	C_21_H_20_O_12_	463.0882	1.30	301, 271
29	Myricetin	5.01	[M−H]^−^	C_15_H_10_O_8_	317.031	4.10	151
30	Naringenin glucoside	5.14	[M−H]^−^	C_21_H_22_O_10_	433.1138	0.92	271, 151, 119
31	Narigenin *	5.31	[M+H]^+^	C_15_H_12_O_5_	273.0749	−5.13	153, 119
32	Kaempferol rutinoside	5.71	[M−H]^−^	C_27_H_30_O_15_	593.1507	0.17	285
33	Luteolin *	5.84	[M+H]^+^	C_15_H_10_O_6_	287.0546	−3.14	153
34	Quercetin *	5.86	[M−H]^−^	C_15_H_10_O_7_	301.0354	1.99	178, 151
35	Kaempferol coumaroyl hexose	5.86	[M+H]^+^	C_30_H_26_O_13_	595.1457	1.01	309, 287
36	Eriodictyol	5.89	[M+H]^+^	C_15_H_12_O_6_	289.0695	−5.88	153, 135
37	Kaempferol	6.01	[M+H]^+^	C_15_H_10_O_6_	287.0552	−1.05	165, 153
38	Naringin	6.62	[M−H]^−^	C_27_H_32_O_14_	579.1710	−0.52	459, 271
39	Apigenin *	6.69	[M−H]^−^	C_15_H_10_O_5_	269.0468	6.69	151, 117
40	Isorhamnetin	7.17	[M−H]^−^	C_16_H_12_O_7_	315.0532	8.89	300, 151
	*Ellagic acid derivatives*						
41	Ellagic acid hexose	3.76	[M−H]^−^	C_20_H_16_O_13_	463.0513	0.22	301
42	Ellagic acid pentose	4.07	[M−H]^−^	C_19_H_14_O_12_	433.0427	4.62	301
43	Ellagic acid deoxyhexose	4.19	[M−H]^−^	C_20_H_16_O_12_	447.058	3.80	301
44	Ellagic acid	4.34	[M−H]^−^	C_14_H_6_O_8_	300.9994	3.32	257, 229
45	Methylellagic acid	4.95	[M−H]^−^	C_15_H_8_O_8_	315.0151	3.49	300
46	Dimethylellagic acid	6.18	[M−H]^−^	C_16_H_10_O_8_	329.0295	−0.61	314, 299
47	Dimethylellagic acid	6.31	[M−H]^−^	C_16_H_10_O_8_	329.0297	0.00	314, 299
48	Trimethylellagic acid	7.88	[M−H]^−^	C_17_H_12_O_8_	343.0456	0.87	328, 299, 284
	*Gallic acid derivatives*						
49	Galloylglucose	0.72	[M−H]^−^	C_13_H_16_O_10_	331.0655	−3.02	169, 125
50	Gallic acid	2.39	[M−H]^−^	C_7_H_6_O_5_	169.0134	−1.78	125
51	Digalloyl glucose	3.11	[M−H]^−^	C_20_H_20_O_14_	483.0766	−1.66	465, 331, 313, 169
52	Digalloyl glucose	3.33	[M−H]^−^	C_20_H_20_O_14_	483.0781	1.45	465, 331, 313, 169
53	Ttrigalloyl glucose	3.69	[M−H]^−^	C_27_H_24_O_18_	635.087	−2.20	483, 465, 331, 313
54	Trigalloyl glucose	3.70	[M−H]^−^	C_27_H_24_O_18_	635.0908	3.78	483, 465, 331, 313
55	Tetragalloyl glucose	4.15	[M−H]^−^	C_34_H_28_O_22_	787.1012	2.29	635, 617, 483, 465, 447, 331
56	Tetragalloyl glucose	4.16	[M−H]^−^	C_34_H_28_O_22_	787.0999	0.64	635, 617, 483, 465, 447, 331
	*Amino acids*						
57	Asparagin *	0.50	[M−H]^−^	C_4_H_8_N_2_O_3_	131.0455	−0.76	114, 95, 70
58	Arginine *	0.54	[M+H]^+^	C_6_H_14_N_4_O_2_	175.1186	−5.14	158, 130, 116
59	Proline *	0.54	[M+H]^+^	C_5_H_9_NO_2_	116.0714	2.24	70
60	Glutamate *	0.54	[M−H]^−^	C_5_H_9_NO_4_	146.0448	−3.42	128, 102
61	Betaine *	0.57	[M+H]^+^	C_5_H_11_NO_2_	118.0858	−8.47	58, 59
62	Glutamine *	0.55	[M+H]^+^	C_5_H_10_N_2_O_3_	147.0758	−7.48	102, 84
63	Phenylalanine *	2.81	[M+H]^+^	C_9_H_11_NO_2_	166.0860	−4.82	120, 103
64	Tryptophan *	3.32	[M+H]^+^	C_11_H_12_N_2_O_2_	205.097	−3.41	188, 170, 118
	*Organic acids*						
65	Fumaric acid *	0.42	[M−H]^−^	C_4_H_4_O_4_	115.0032	0.87	71
66	Citric acid *	0.7	[M−H]^−^	C_6_H_8_O_7_	191.0198	3.66	111, 87, 85
67	Malic acid *	0.7	[M−H]^−^	C_4_H_6_O_5_	133.0133	−3.01	115, 89, 71
	*Phenolic acid*						
68	Coumaric acid *	0.39	[M−H]^−^	C_9_H_8_O_3_	163.0385	−6.13	119, 117, 93
69	Caffeic acid *	0.4	[M−H]^−^	C_9_H_8_O_4_	179.0348	2.23	135, 134, 107
70	Quinic acid *	0.61	[M−H]^−^	C_7_H_12_O_6_	191.055	−2.62	173, 127, 93, 85
71	Salicylic acid *	3.59	[M+H]^+^	C_7_H_6_O_3_	139.0387	−5.75	121, 93
72	Ferulic acid	3.84	[M+H]^+^	C_10_H_10_O_4_	195.0646	−5.64	177
73	Phloretin	6.6	[M−H]^−^	C_15_H_14_O_5_	273.0777	5.13	179, 167

* Identified by standard substances. ^a^ GC: (epi)gallocatechin; C: (epi)catechin; C(G): (epi)catechin gallate.

**Table 2:** Total phenol and free radical scavenging activity of whole *C. crenata* shell extracts.

Samples	Total Phenol Content (mg GAE/g) ^a^	DPPH Free Radical Scavenging Activity IC_50_ (mg/L)	FRAP Value (mmol Fe/g Dry Weight) (%)
Okkwang	32.57 ± 6.06	42.23 ± 9.61	6.70 ± 1.28
Daebo	25.32 ± 5.77	53.76 ± 16.10	5.16 ± 1.19
Riheiguri	22.27 ± 7.38	57.52 ± 13.11	4.65 ± 1.42
Porotan	35.55 ± 3.91	40.29 ± 5.69	7.35 ± 1.13
Ishizuuchi	44.80 ± 8.59	29.64 ± 9.83	9.23 ± 1.78

Data are presented as mean ± SD. ^a^ Expressed as milligrams of GAE per gram of extract.

## Data Availability

Not applicable.

## Supplementary Materials

The following supporting information can be downloaded at: https://www.mdpi.com/article/10.3390/biom12121797/s1, Figure S1: Morphological features of five *C. crenata* cultivars; Figure S2: Total ion chromatograms in (A) ESI-positive and (B) ESI-negative modes from UPLC-QTOF/MS of whole *C. crenata* shells; Figure S3: PLS-DA score plot derived from UPLC-QTOF/MS spectra of whole *C. crenata* shells extracts. (A) positive, (B) negative mode; Table S1: Sample list of *C. crenata* analyzed in the present study; Table S2: Retention times and MRM transitions of metabolites quantified from *C. crenata* shells by LC-QTRAP/MS; Table S3: The list of compounds in heatmap visualization; Table S4: LC-QTRAP/MS-MRM calibration curve equations and linear correlation coefficients (*R*^2^) for metabolites quantified in *C. crenata* shells.
