# Supplementary material for: Metabolic Profiling of Chestnut Shell (Castanea crenata) Cultivars Using UPLC-QTOF-MS and Their Antioxidant Capacity
Source: Biomolecules. 2022 Dec 1;12(12):1797. doi: 10.3390/biom12121797 (PMC9775926; doi:10.3390/biom12121797)
Supplement: Supplementary file 1 [file biomolecules-12-01797-s001.zip › biomolecules-2065734-supplementary.pdf]

Table S1. Sample list of *C. crenata* analyzed in the present study.

Region	Number of samples	Cultivar name	Origin	Crossbreeding
Chungchengnam-do (latitude 36° N, Longitude 127° E)	22	Okkwang	Korea	*crenata* [a]
	11	Daebo	Korea	*crenata* hybrid (Sangmyeon 1 [a] × Riheiguri)
	12	Ishizuuchi	Japan	*crenata* hybrid (Ganne [b] × Kasaharawase [b])
	15	Porotan	Japan	(*crenata* × *bungeana*) × *crenata* hybrid (550-40 × Tanzawa [b])
	14	Riheiguri	Japan	*crenata* × *mollissima* hybrid

[a] The origin of cultivar was Korea only. [b] The origin of cultivar was Japan only.

Table S2. Retention times and MRM transitions of metabolites quantified from *C. crenata* shells by LC-QTRAP/MS.

**Compound**	**Retention time (min)**	**Precursor ion (*m/z*)**	**Collision energy (eV)**	**MRM ion transitions (*m/z*)**
Shikimic acid	0.99	172.8	-20	93.0
Gallic acid	3.88	168.8	-23	125.0
Tryptophan	4.59	202.9	-24	159.0
Chlorogenic acid	5.22	353.1	-29	191.0
Catechin	5.26	288.9	-31	245.0
Caffeic acid	5.62	178.9	-25	135.0
Rutin	5.98	609.2	-62	299.9
Quercetin glucose	6.20	463.1	-40	300.0
Coumaric acid	6.24	162.8	-22	119.0
Ferulic acid	6.56	192.9	-22	134.0
Luteolin	7.67	284.8	-55	132.9
Quercetin	7.72	300.9	-36	150.9
Naringenin	8.25	270.9	-34	150.9
Apigenin	8.25	268.9	-49	116.9
Phenylalanine-$^{13}C_6$ (IS)	4.06	169.9	-20	153.2

Table S3. The list of compounds in heatmap visualization.

Group	Class	Compound	Comp no.
1	Proanthocyanidins	C-C-C B-type trimer	19
	Proanthocyanidins	C(G)-C B-type dimer	20
	Ellagitannins	galloyl-HHDP-glucose	2
	Flavonoids	Luteolin	33
	Flavonoids	Kaempferol	37
	Ellagic acid derivatives	Dimethylellagic acid	46
	Ellagic acid derivatives	Trimethylellagic acid	48
	Gallic acid derivetives	Tetragalloyl glucose	55
	Flavonoids	Quercetin	34
	Flavonoids	Isorhamnetin	40
2	Flavonoids	Naringin	38
	Flavonoids	Kaempferol-rutinoside	32
	Flavonoids	Kaempferol coumaroyl hexose	35
	Ellagitannins	Trigalloyl-HHDP-glucose	9
	Gallic acid derivetives	Digalloyl glucose	52
	Ellagitannins	Digalloyl-HHDP-glucose	8
	Gallic acid derivetives	Trigalloyl glucose	54
	Ellagic acid derivatives	Ellagic acid deoxyhexose	43
	Flavonoids	Rutin	27
	Flavonoids	Apigenin	39
	Ellagic acid derivatives	Ellagic acid hexose	41
	Ellagic acid derivatives	Ellagic acid pentose	42
	Ellagitannins	Bis-HHDP-glucose	5
	Ellagitannins	HHDP-valoneoyl-glucose	6
	Ellagitannins	HHDP-glucose	1
	Ellagitannins	NHTP-HHDP-glucose	3
3	Proanthocyanidins	C-C B-type dimer	18
	Flavonoids	Catechin	25
	Phenolic acid	Salicylic acid	71
	Flavonoids	Eriodictyol	36
	Phenolic acid	Phloretin	73
	Flavonoids	Epigallocatechin	24
	Proanthocyanidins	GC-GC-C B-type trimer	11
	Proanthocyanidins	GC-GC B-type dimer	14
	Flavonoids	Naringenin glucoside	30
	Flavonoids	Narigenin	31
	Flavonoids	Myricetin-hexoside	26

	Flavonoids	Quercetin hexose	28
	Organic acids	Citric acid	66
	Phenolic acid	Quinic acid	70
	Amino acids	Phenylalanine	63
	Amino acids	Tryptophan	64
4	Phenolic acid	Ferulic acid	72
	Gallic acid derivetives	Galloylglucose	49
	Gallic acid derivetives	Gallic acid	50
	Amino acids	Glutamate	60
	Organic acids	Malic acid	67
	Amino acids	Arginine	58
	Amino acids	Asparagin	57
	Amino acids	Proline	59
	Amino acids	Betaine	61
	Amino acids	Glutamine	62
	Ellagic acid derivatives	Ellagic acid	44
	Organic acids	Fumaric acid	65
	Phenolic acid	Coumaric acid	68
	Phenolic acid	Caffeic acid	69
	Ellagic acid derivatives	Methylellagic acid	45
	Proanthocyanidins	GC-GC-GC B-type trimer	16
	Flavonoids	Myricetin	29

Table S4. LC-QTRAP/MS-MRM calibration curve equations and linear correlation coefficients (R^2) for metabolites quantified in *C. crenata* shells.

Compounds	Con. range (nmol/L)	Calibration equations	R^2
Shikimic acid	10-1000	y = 0.0401x – 0.8948	0.9965
Gallic acid [a]	6-600	y = 3.837x + 8.185	0.9973
Tryptophan [a]	0.05-10	y = 4.652x + 1.941	0.9944
Chlorogenic acid	0.5-50	y = 2.052x – 0.6847	0.9953
Catechin [a]	0.15-15	y = 3.014x + 1.604	0.9970
Caffeic acid	0.3-30	y = 1.821x + 2.451	0.9941
Rutin	10-1000	y = 1.603x + 1.490	0.9911
Quercetin glucose	2-200	y = 1.235x + 3.106	0.9988
Coumaric acid	10-1000	y = 1.615x + 0.0554	0.9987
Ferulic acid	6-600	y = 0.3190x – 1.203	0.9919
Luteolin	10-1000	y = 1.975x + 3.053	0.9957
Quercetin [a]	0.1-10	y = 7.119x + 0.3907	0.9978
Naringenin	10-1000	y = 3.581x + 0.0042	0.9995
Apigenin	1.5-150	y = 1.674x – 0.5507	0.9973

[a] The concentration is μmol/L.

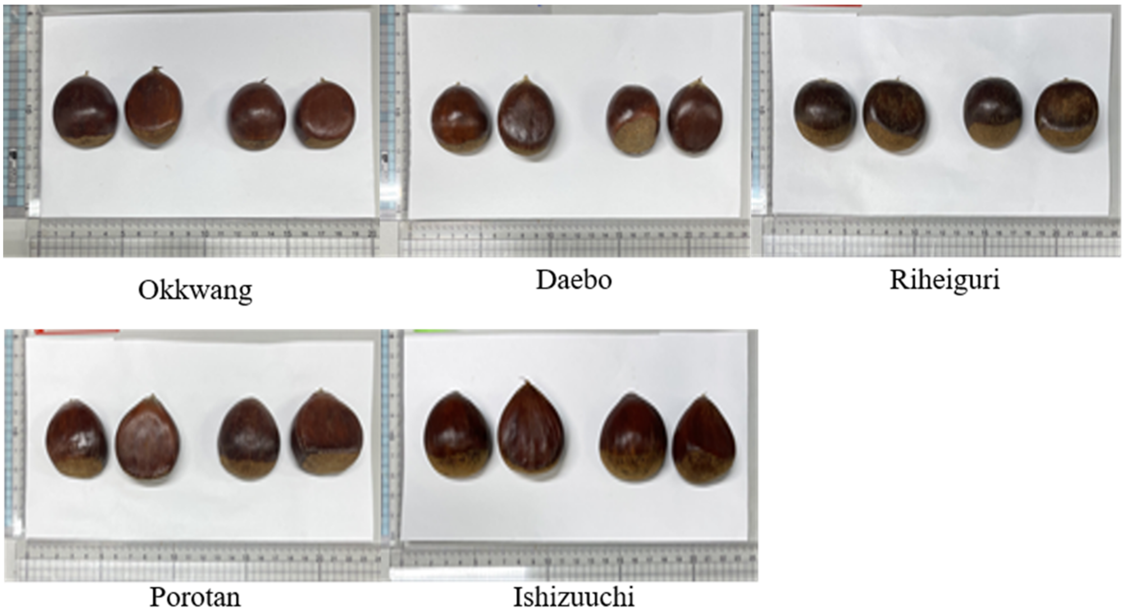

Figure S1. Morphological features of five *C. crenata* cultivars.

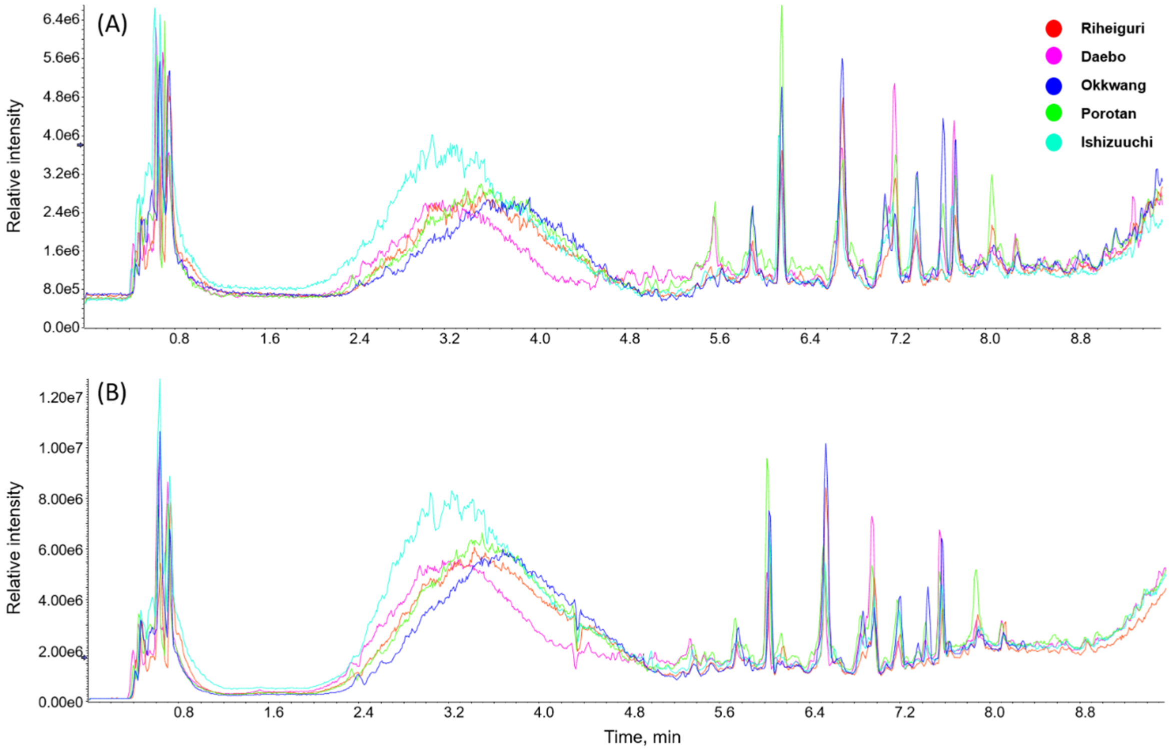

Figure S2. Total ion chromatograms in (A) ESI-positive and (B) ESI-negative modes from UPLC-QTOF/MS of whole *C. crenata* shells .

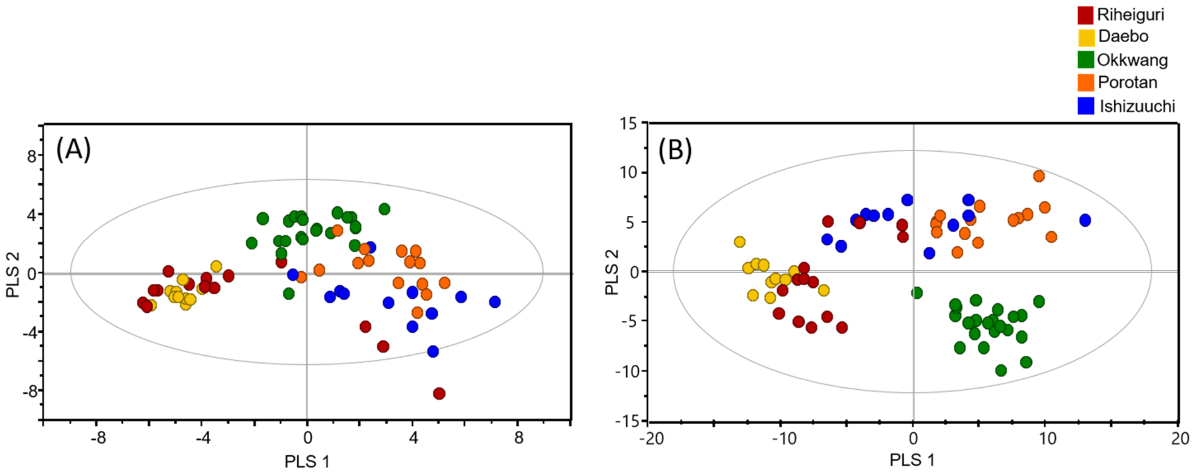

Figure S3. PLS-DA score plot derived from UPLC-QTOF/MS spectra of whole *C. crenata* shells extracts. (A) positive, (B) negative mode.
